# Association of Peripheral Monocyte Count with Soluble P-Selectin and Advanced Stages in Nasopharyngeal Carcinoma

**DOI:** 10.1155/2018/3864398

**Published:** 2018-03-05

**Authors:** Adi Surya Komala, Andhika Rachman

**Affiliations:** Internal Medicine Department, Faculty of Medicine, Universitas Indonesia, Salemba Raya 6, Central Jakarta 10430, Indonesia

## Abstract

**Introduction:**

Inflammation is widely recognized to play an important role in cancer progression and is related to thrombosis. Soluble P-selectin (sP-selectin) is one of several biomarkers that may be predictive of thrombosis in cancer. This study aimed to investigate the correlation between monocyte count and sP-selectin in various stages of nasopharyngeal carcinoma.

**Methods:**

Fifty-five patients with nasopharyngeal carcinoma were divided into three groups according to nodal and distant metastasis (group of stages I-IVA, IVB, and IVC). Monocyte count was calculated from routine peripheral blood examination, while sP-selectin level was measured using commercial ELISA kit.

**Results:**

The monocyte count of subjects in groups IVB and IVC was significantly higher compared to group I-IVA (707/*μ*L versus 528/*μ*L, *p* = 0.022; 841/*μ*L versus 528/*μ*L, *p* = 0.005). Plasma levels of sP-selectin in group IVC were higher than group I-IVA (59.5 ng/mL versus 41.97 ng/mL, *p* = 0.001) and group IVB (59.5 ng/mL versus 45.53 ng/mL, *p* = 0.007). In subjects with high monocyte count (>665/*μ*L), there was moderate correlation between monocyte count and sP-selectin (*r* = 0.436, *p* = 0.022).

**Conclusion:**

Advanced stages of nasopharyngeal carcinoma had higher levels of monocyte count and sP-selectin compared to earlier stages. Monocyte count was correlated with sP-selectin especially in high monocyte count subgroup.

## 1. Introduction

Nasopharyngeal carcinoma is a unique malignancy showing a distinct racial and geographical distribution [1]. This type of malignancy is endemic in Southern China and the incidence is up to 25 per 100,000 persons. Intermediate incidence is seen in Southeast Asia, the Mediterranean, and the Arctic [2]. In Indonesia, nasopharyngeal carcinoma is the fourth most common cancer after cervical, breast, and skin cancer with an incidence of 5.66 per 100,000 persons [3].

The tumor-node-metastasis (TNM) classification is a worldwide benchmark for reporting the extent of malignant disease and is a major prognostic factor in predicting the outcome of patients with cancer [4]. A number of studies have shown that the T (tumor) stage was no longer being the most influential factor of treatment outcomes in nasopharyngeal carcinoma. The overall survival rates of stages I, II, III, and IVA were not significantly different, suggesting the N stage as the key prognostic factor [5].

Cancer and its treatment are well-recognized risk factors for thrombosis [6]. Overall the incidence of thrombosis in cancer patients might be as high as 60% [7] that represents the second leading cause of death in hospitalized patients with cancer [8]. Many factors have been shown to be risk factors for thrombosis, including cancer site. Compared to other sites, head and neck cancer relatively had high incidence of venous thromboembolism [9]. Platelet count, leukocyte count, hemoglobin, d-dimer, tissue factor, soluble P-selectin, factor VIII, and prothrombin fragment F 1+2 are potential biomarkers that may be predictive of vein thromboembolism in cancer [10–12].

Inflammation has also been known to be related to both arterial and venous thrombosis [13, 14]. The pathogenesis of inflammation-induced thrombosis is complicated [14]. Multiple mechanisms are at play including up regulation of the tissue factor leading to the initiation of clotting, amplification of the clotting process by augmenting exposure of cellular coagulant phospholipids, inhibition of fibrinolysis by elevating plasminogen activator inhibitor 1 (PAI-1), and depletion of natural anticoagulant, particularly targeted toward downregulation of the protein C anticoagulant pathway [15].

Inflammation is widely recognized to play an important role in cancer progression [16–19] and is proposed as the seventh cancer hallmark [20]. Epidemiological studies have revealed that chronic inflammation predisposes to different forms of cancer [21]. Regular usage of aspirin or other traditional nonsteroidal anti-inflammatory drugs is associated with a reduced risk of developing cancer [22], mirroring the finding of inflammation as a risk factor for cancers [21]. Many similarities can be found in the microenvironment of chronic inflammatory conditions and tumors, suggesting a role for inflammatory cells and cytokines in tumors progression and immunosuppression [13].

Monocytes are members of the mononuclear phagocyte system that comprises monocytes, dendritic cells, and macrophages. After birth, monocytes will enter the blood circulation and be recruited into tissues through the body. During inflammation, monocytes will become the dominant mononuclear phagocytes present in inflamed tissues and lymph nodes [23]. In cancer, tumor-derived factors will cause sustained myelopoiesis, accumulation, and functional differentiation of myelomonocytic cells [24]. A high monocytes count has been independently associated with the prognosis of various malignancies, such as gastric cancer, acute lymphoblastic leukemia, lymphoma, hepatocellular carcinoma, and nasopharyngeal carcinoma [25]. In this study, we wanted to evaluate the correlation between monocyte count, soluble P-selectin (sP-selectin), and the cancer stage in nasopharyngeal carcinoma, one of the most cancer types in Indonesia.

## 2. Materials and Methods

### 2.1. Patient Selection

In this study, nasopharyngeal carcinoma patients admitted to Cipto Mangunkusumo Hospital, Jakarta, Indonesia, from April to November 2012, were included if meeting the following criteria: pathologically proven nasopharyngeal carcinoma according to World Health Organization (WHO) histological classification (type I, keratinizing squamous cell carcinoma; type II, differentiated nonkeratinizing squamous cell carcinoma; type III, undifferentiated nonkeratinizing squamous cell carcinoma), no prior malignancy, no previous chemotherapy or radiotherapy, no concurrent infections (including hepatitis B and hepatitis C), no diabetes, no heart failure, no kidney diseases, and without any antithrombotic therapy. This study was approved by the ethics committees of Cipto Mangunkusumo Hospital.

### 2.2. Blood Tests

Blood samples were collected from each patient in EDTA and citrate anticoagulant treated tubes. Samples from EDTA treated tubes were analyzed for routine peripheral blood cells (e.g., hemoglobin levels, lymphocytes, neutrophils, eosinophils, basophils, monocytes, and platelets) using a Sysmex XE-2100 automated hematology system. Plasma level of sP-selectin was measured using commercially available ELISA kit (R&D systems, Minneapolis, USA) according to a previously described method [26]. Samples were obtained after having informed consent and stored at −80°C until analysis.

### 2.3. Statistical Analysis

Analysis of variance was used to compare sP-selectin levels between several stages of nasopharyngeal carcinoma. The bivariate correlations procedure from SPSS 13 was used to compute Spearman's *ρ* correlation coefficient for monocyte count and sP-selectin level. *p* values less than 0.05 were accepted as significant.

## 3. Result

### 3.1. Patients' Characteristics

Fifty-five patients were included in this study. The basic patients' characteristics are shown in Table 1. The median age at the time of diagnosis was 43 years and 76% of patients were male. Almost all patients had advanced stages and 31% had distant metastases at presentation. The bones (76%) were the most common sites of metastasis, followed by the liver (29%) and lung (24%).

### 3.2. Monocyte Count and Soluble P-Selectin Levels in Different Stages

All of the subjects were further grouped into three groups according to nodal and distant metastasis, group of stages I-IVA (T*x*N0–N2, M0), IVB (T*x*N3M0), and IVC (T*x*N*x*M1) (Table 2).

**Figure 1:**
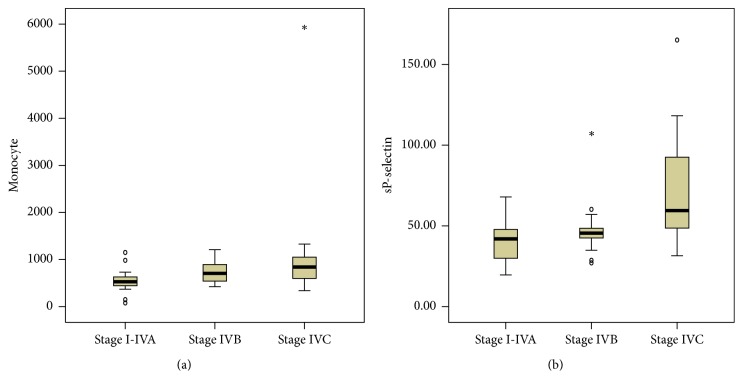
Comparison of monocyte count (a) and sP-selectin (b) between groups.

Median levels of monocyte count in groups IVB and IVC were significantly higher compared to group I-IVA (707/*μ*L [540–913] versus 528/*μ*L [441–663], *p* = 0.022; 841/*μ*L [587–1065] versus 528/*μ*L [441–663], *p* = 0.005) (Figure 1(a)). Statistically there was no difference between the median level of monocyte count in group IVC and IVB (841/*μ*L [587–1065] versus 707/*μ*L [540–913], *p* = 0.261) (Table 3).

Subjects in group IVC had a higher sP-selectin compared to group I-IVA and IVB (59.5 ng/mL [46.98–93.3] versus 41.97 ng/mL [28.12–48.23], *p* = 0.001; 59.5 ng/mL [46.98–93.3] versus 45.53 ng/mL [42.24–54.41], *p* = 0.007). Plasma levels of sP-selectin in group IVB were not different with group I-IVA (45.53 ng/mL [42.24–54.41] versus 41.97 ng/mL [28.12–48.23], *p* = 0.194) (Figure 1(b)).

Jiang et al. found that, in metastatic nasopharyngeal carcinoma, an increased monocyte count (≥665/*μ*L) was significantly associated with poor prognosis [27]. By using that cut-off, we tried to separate the subjects into two categories, <665/*μ*L and ≥665/*μ*L groups. Subjects with higher monocyte count (≥665/*μ*L) had significant higher sP-selectin levels compared to lower monocyte count (<665/*μ*L) (47.39 ng/mL [43.41–71.34] versus 44.98 ng/mL [32.94–52.91], *p* = 0.023) (Table 4).

### 3.3. Correlation of Monocyte and Platelet Count with sP-Selectin

Platelet count had no correlation with sP-selectin (*p* = 0.213), whereas monocyte count had a weak correlation with sP-selectin (*r* = 0.379, *p* = 0.004). In subjects with higher monocyte count (>665/*μ*L), there was moderate correlation between monocyte count and sP-selectin (*r* = 0.436, *p* = 0.022).

## 4. Discussion

Nasopharyngeal carcinoma has a unique and complex etiology that is not completely understood. Although its incidence is low in most parts of the world, it is endemic in a few well defined populations, including natives of Southern China, Southeast Asia, Arctic, and the Middle East/North Africa [2, 28]. In Indonesia, the incidence of nasopharyngeal carcinoma is around 5.66/100000 people [3]. More than 30% of subjects in this study had advanced stage at presentation and bones became the most common site of metastasis. Once metastasis is diagnosed, the overall survival of patients is typically under 15 months with palliative chemotherapy [27].

Thrombosis, particularly venous thromboembolism (VTE) is a frequent complication in cancer and occurs in up to 20% of cancer patients [29]. The VERITY study revealed that certain tumor sites were common in VTE cases compared to non-VTE cases including central nervous system, pancreas, upper gastrointestinal tract, and head/neck. In that study, head and neck cancers (including nasopharyngeal carcinoma) had the second highest incidence of VTE (odds ratio of 8.24) [9]. Until now, there is no prospective observational data showing the incidence of thrombosis in nasopharyngeal carcinoma. Thrombotic complications significantly contribute to morbidity and mortality in cancer [30]. Preventing these complications is clinically relevant [24] and several parameters have been proposed as potential biomarkers of increased risk of VTE.

Soluble P-selectin as well as tissue factor, D-dimer, C-Reactive Protein, platelet, and leukocyte counts has been proposed as promising biomarkers that may be predictive of VTE in cancer [12]. Data from the Vienna Cancer and Thrombosis Study (CATS) showed that high sP-selectin levels (cut-off level, 53.1 ng/mL) could independently predict VTE in cancer patients (HR = 2.6; 95% CI, 1.4 to 4.9) [11]. Previous multiple studies had also shown an increased risk of VTE in patients with advanced stage of cancer [31].

P-selectin is an adhesion molecule constitutively expressed in the *α*-granules of platelets that will be shed as soluble P-selectin following platelets activation [32]. Although it is also expressed in the Weibel-Palade bodies of endothelial cells, platelets are thought to be the major source of circulating sP-selectin in healthy individuals [33]. In contrast with previous reports, Riedl et al. in their nested case-control study within a cohort of 1779 patients with different types of cancer suggest that high levels of sP-selectin in cancer patients might derive from a distinct source other than platelets [29]. They obtained weak correlation between platelet count and sP-selectin (*r* = 0.105, *p* = 0.039) which was not found in our study (*p* = 0.213). It could be due to the limited number of subjects in this study.

We found that the mean levels of sP-selectin were 52.81 ng/mL and the median level of sP-selectin in patients with distant metastasis was 59.5 ng/mL putting them in a high risk of thrombosis. This level was statistically different compared to the sP-selectin levels of other groups (*p* < 0.05) (Table 3). We then separated the subjects without distant metastasis according to the extent of nodal metastasis and there was no difference in sP-selectin levels between group IVB (regional lymph nodes N3) and group I-IVA (N0–N2). Less interaction of tumor cells with platelets or endothelial cells in nondistant metastasis group could be the reason for this result.

Inflammation has significant contributions in cancer progression. Monocytes as part of mononuclear phagocyte system play a key role not only in inflammation but also in cancer. Tumor-derived factors will attract circulating monocytes into the tumor tissue where they differentiate into macrophages (tumor associated macrophages (TAM)). This differentiation process is accompanied by vast phenotypic, functional, and morphological changes, establishing a spectrum of diverse macrophage populations throughout the body [34]. As in other tumor types, a high density of TAM in the tumor stroma correlates with poor prognosis in nasopharyngeal carcinoma [35].

Tumor-derived lymphangiogenic growth factors (especially Vascular Endothelial Growth Factor-C (VEGFC)) can drive the formation of lymphatic vessels, providing an interface for lymphatic vessel invasion and distant metastasis [36]. Not only for lymphatic vessels, VEGFC is also a chemoattractant in mobilizing monocytes from their pool in the bone marrow and spleen [37]. In nasopharyngeal carcinoma, Li et al. found that the 5-year overall survival Hazard Ratio (HR) of patients with monocyte count ≥ 475/*μ*L compared to <475/*μ*L was 1.409 (95% CI, 1.078–1.843) [25]. Another study by Jiang found that monocyte count ≥ 665/*μ*L was an independent prognostic factor for patient with metastatic nasopharyngeal carcinoma (HR = 1.98; 95% CI, 1.63–2.41) [27]. Associated with these findings, we found that monocyte count in patient with groups IVC (distant metastasis) and IVB (Node N3) was not different although their levels were higher than other group. Compared to other tumor types, head and neck cancer is a group of cancer that has strong correlation between lymph node metastasis and distant metastasis [38]. It could explain why there was no difference in monocyte count between group IVC and IVB.

Monocyte, alongside neutrophils and platelets, was known to be responsible for the initiation and amplification of deep vein thrombosis [39]. Monocyte participates in the prothrombotic condition directly via the secretion of procoagulant factors and indirectly by promoting inflammation process. The adhesion of leukocyte to platelets deposited at the sites of vascular injury may represent an important mechanism by which leukocytes contribute to haemostasis and thrombosis [40]. It was further supported by the observation that monocytes constituted 16% of platelet-bound leukocytes in vascular thrombosis. In vivo study of venous thrombosis in mice by von Bruhl et al. demonstrated that 30% of all accumulated leukocytes within venous thrombi were monocytes [39].

Cafolla found a lower level of monocyte count in patients having partial resolution of thrombosis compared to the time at diagnosis [41]. Our study found that monocyte count had a weak correlation with sP-selectin (*r* = 0.379, *p* = 0.004). If we divided the patients using the monocyte count cut-off from Jiang, there were significant levels of sP-selectin in higher monocyte count patients (47.39 ng/mL [43.41–71.34] versus 44.98 ng/mL [32.94–52.91], *p* = 0.023) (Table 4). We also found an increased correlation between monocyte count and sP-selectin in higher monocyte count patients (*r* = 0.436, *p* = 0.022). In agreement with Cafolla, we suggest that monocyte count could be a possible marker for predicting the possibility of having thrombosis in patient with cancer.

Not being an observational prospective study and not exploring the thrombotic events in all subjects are the main limitations in this study. The plasticity of monocyte between circulating subsets might also affect the accuracy of monocyte count. Not obtaining samples with special anticoagulants that inhibit ex vivo platelet activation is another limitation.

## 5. Conclusion

Patients with advanced stages of nasopharyngeal carcinoma had higher levels of monocyte count and sP-selectin than patients with earlier stages. Monocyte count was correlated with sP-selectin, a biomarker of venous thromboembolism, and the correlation was increased in high monocyte count subgroup. Further observational prospective studies need to confirm the importance of monocyte count and sP-selectin as thrombosis markers in nasopharyngeal carcinoma.

## Figures and Tables

**Table 1:** Patients' characteristics.

Characteristic	Case (%) (*n* = 55)
Gender	
Female	13 (23.6%)
Male	42 (76.4%)
Age (mean)	43.84 ± 12.03
Age group	
<20 year	2 (3.6%)
21–30 year	4 (7.3%)
31–40 year	16 (29.1%)
41–50 year	14 (25.5%)
51–60 year	14 (25.5%)
>60 year	5 (9.1%)
Stage (TNM classifications)	
I	1 (1.8%)
II	1 (1.8%)
III	3 (5.5%)
IVA	14 (25.5%)
IVB	19 (34.5%)
IVC	17 (30.9%)
Pathology (WHO)	
Type I	1 (1.8%)
Type II	9 (16.4%)
Type III	45 (81.8%)

**Table 2:** Comparison of laboratory parameters between groups.

	Group of stage		
	I-IVA	IVB	IVC
	(*n* = 19)	(*n* = 19)	(*n* = 17)
Hemoglobin (g/dL)	13.27 ± 1.57	13.09 ± 1.42	10.73 ± 1.92
Leukocyte (/*μ*L)	8414.74 ± 2770.30	9588.95 ± 3641.2	15697.06 ± 14326.49
Platelet (/*μ*L)	3250000 ± 89290.41	316631.58 ± 69952.53	330117.65 ± 157547.2
Monocyte (/*μ*L)	545.11 ± 246.43	734.32 ± 235.88	1120.94 ± 1271.41
sP-selectin (ng/mL)	40.88 ± 14.12	47.89 ± 16.44	71.65 ± 33.70

**Table 3:** Comparison of monocyte count and sP-selectin between groups.

Group	*n*	Monocyte	*p*	sP-selectin	*p*
		Median		Median	
		(25th–75th percentile)		(25th–75th percentile)	
I-IVA	19	528 (441–663)		41.97 (28.12–48.23)	
IVB	19	707 (540–913)	0.008	45.53 (42.24–50.41)	0.001
IVC	17	841 (587–1065)		59.5 (46.98–93.3)	

**Table 4:** Comparison of sP-selectin levels based on monocyte count groups.

Monocyte count	*n*	sP-selectin		*p*
		Mean	Median (25th–75th percentile)	
<665/*μ*L	28	44.20	44.98 (32.94–52.91)	0.023
≥665/*μ*L	27	61.74	47.39 (43.41–71.34)	
